# Study of Cathepsin B inhibition in VEGFR TKI treated human renal cell carcinoma xenografts

**DOI:** 10.1038/s41389-019-0121-7

**Published:** 2019-02-22

**Authors:** Chun-Hau Chen, Swati Bhasin, Prateek Khanna, Mukta Joshi, Patrick MN. Joslin, Ruchi Saxena, Seema Amin, Suhu Liu, Shreya Sindhu, Sarah R. Walker, Paul Catalano, David A. Frank, Seth L. Alper, Manoj Bhasin, Rupal S. Bhatt

**Affiliations:** 1 Department of Medicine, Beth Israel Deaconess Medical Center, Harvard Medical School, Boston, MA 02215 USA; 2 Department of Medicine, Division of Interdisciplinary Medicine and Biotechnology, Beth Israel Deaconess Medical Center, Harvard Medical School, Boston, MA 02115 USA; 3 BIDMC Genomics, Proteomics, Bioinformatics and Systems Biology Center, Beth Israel Deaconess Medical Center, Boston, MA 02115 USA; 4 Department of Medical Oncology, Dana-Farber Cancer Institute, Department of Medicine, Brigham and Women’s Hospital and Harvard Medical School, Boston, MA 02215 USA; 5 Department of Data Sciences, Dana-Farber Cancer Institute, Department of Biostatistics, Harvard T.H. Chan School of Public Health, Boston, MA USA

## Abstract

Several therapeutic options are available for metastatic RCC, but responses are almost never complete, and resistance to therapy develops in the vast majority of patients. Consequently, novel treatments are needed to combat resistance to current therapies and to improve patient outcomes. We have applied integrated transcriptome and proteome analyses to identify cathepsin B (CTSB), a cysteine proteinase of the papain family, as one of the most highly upregulated gene products in established human RCC xenograft models of resistance to vascular endothelial growth factor receptor (VEGFR) tyrosine kinase inhibitors (TKI). We used established RCC models to test the significance of CTSB in the progression of renal cancer. Our evaluation of CTSB showed that stable CTSB knockdown suppressed RCC growth in vitro and in vivo. Stable over-overexpression of wild-type CTSB (CTSB^wt/hi^), but not of an CTSB active site mutant (CTSB^N298A^), rescued cell growth in CTSB knockdown cells and abolished the efficacy of VEGFR TKI treatment. Genome-wide transcriptome profiling of CTSB knockdown cells demonstrated significant effects on multiple metabolic and stem cell-related pathways, with ALDHA1A (ALDH1) as one of the most significantly downregulated genes. Importantly, survival analysis across 16 major TCGA cancers revealed that CTSB overexpression is associated with low rates of three and five year patient survival rates (*P* = 2.5e–08, HR = 1.4). These data strongly support a contribution of CTSB activity to RCC cell growth and tumorigenicity. They further highlight the promise of CTSB inhibition in development of novel combination therapies designed to improve efficacy of current TKI treatments of metastatic RCC.

## Introduction

Many patients with metastatic renal cell carcinoma (RCC) benefit from treatment with tyrosine kinase inhibitors (TKIs) such as sunitinib, axitinib, and pazopanib, which act through blockade of vascular endothelial growth factor receptor (VEGFR)^1^. Unfortunately, however, tumors eventually develop resistance to this therapeutic strategy. The VEGFR TKIs likely affect tumor growth through their activity on the cancer’s endothelium, but may also induce substantial changes in tumor cells and their extravascular microenvironment. How tumors survive and grow in the continued presence of VEGF pathway blockade remains a fundamental unanswered question in the field of anti-angiogenic therapy. The advent of novel combinations of anti-angiogenic and immune therapies offers hope for improved patient responses^2^. However, despite these and other research advances, patients continue to experience clinical disease progression, and physicians lack the ability to augment TKI responses in patients who have developed resistance to prior VEGFR therapy. Thus, novel strategies are required to combat resistance toward TKI therapy and to enhance overall survival. We have previously demonstrated the utility for treatment of metastatic mRCC of simultaneous targeting of proteins controlling distinct phases of angiogenesis, such as ALK-1 and VEGFR; this combination therapy results in regression of advanced tumors by downregulating multiple genes from the Notch signaling pathway^3^.

Building on these results, we have developed mouse RCC models of TKI resistance and performed genome-wide transcriptome and proteome analyses to better understand the mechanisms of resistance, as well as to identify their underlying key genetic drivers. Our integrative systems biology analyses identified Cathepsin B (CTSB) as one of the key gene products upregulated in the state of TKI resistance. CTSB is a cysteine proteinase of the papain family normally present in lysosomes, and an important regulator in various pathologies and oncogenic processes. Altered regulation of CTSB expression in the tumor microenvironment may be involved in development of several cancers, and CTSB overexpression correlates with invasive and metastatic phenotypes^4^. CTSB secretion into the tumor’s extracellular microenvironment can initiate several proteolytic cascades, including that leading to TGF-β activation^5^. CTSB also plays an important role in tumor angiogenesis through proteolytic remodeling of the extracellular matrix (ECM), a key step in vessel sprouting during angiogenesis. Moreover, CTSB is an important mediator of apoptosis, with proteolytic activity against several cytosolic caspases and other anti-apoptotic proteins. CTSB has recently been implicated in increased expression of pro-inflammatory cytokines such as interleukin-1-beta (ILβ), monocyte chemotactic protein–1 (MCP1), interleukin-6 (IL-6), and tumor necrosis factor-alpha (TNFα). CTSB also regulates cancer stem cells (CSCs), long implicated in drug resistance and progression of cancer^6^. As true for normal tissue stem cells, this subpopulation of tumor cells is able to initiate repopulation, including the full hierarchy of differentiated cells within a tumor^7^. A hallmark of CSCs is their ability to populate and form new tumors upon serial injection into mice, a property not exhibited by more differentiated cells derived from the same cancer. Thus, tumor cells possessed of the ability to proliferate after isolation from xenografts and transplantation into naive mice are considered CSCs^8^. CSCs contribute to cancer initiation and progression, therapeutic resistance, and metastasis^6,9^, and TKI resistance in RCC patients has been attributed, at least in part, to CSCs. Bussolati and colleagues recently showed that clones of CD105 + tumor/progenitor cells from human renal carcinomas can maintain hemostasis and differentiate into tumor endothelium and epithelium, both in vitro and in vivo^8,10^.

CTSB supports CSC function and maintains tumor cell survival through degradation of ECM, modulation of immune responses, and regulation of autophagy^4,11^. While CTSB has been implicated in oncogenic processes in several types of cancer, to our knowledge there are no published data demonstrating CTSB’s function in RCC^12^. We report here a novel role of CTSB in RCC and a novel mechanism of RCC resistance to VEFGR TKI therapy.

## Results

### Sunitinib resistance of RCC xenografts is associated with altered tumor expression of six proteins

**Fig. 1:**
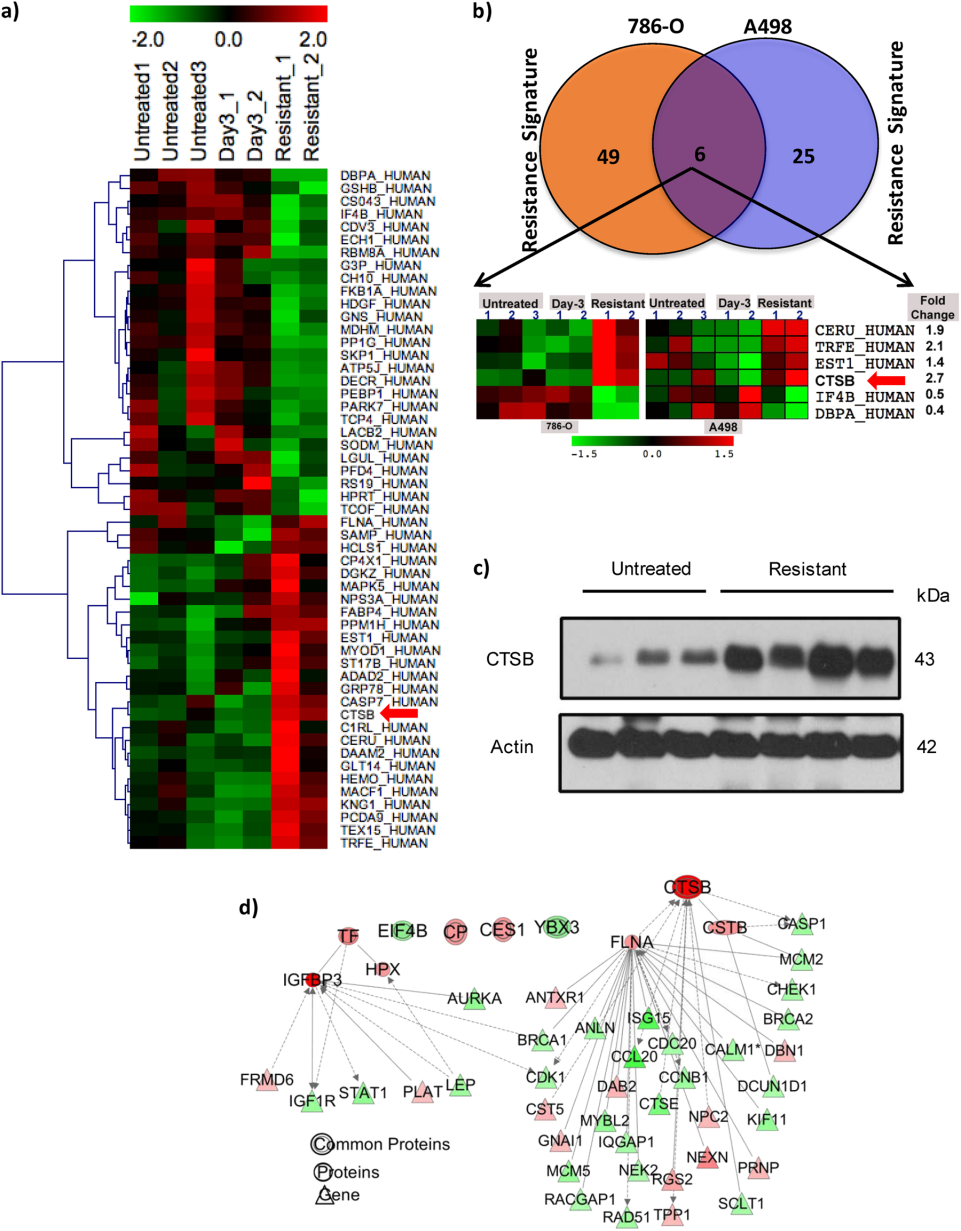
Global proteome analysis of sunitinib-treated resistant tumors. **a** Heatmap of proteins associated with resistance in 786-O RCC xenograft mouse model. Rows depict proteins altered during resistance, and columns depict individual replicates of untreated, day 3, and resistant tumors. Relative protein expression is shown in pseudocolor, where green represents down- regulation, and red represents up-regulation. **b** Venn Diagram (above) and heatmap (below) of proteins whose altered expression is associated with sunitinib resistance in 786-O and A498 RCC xenograft mouse models. Altered expression of six of these proteins (depicted in heatmap) is associated with sunitinib resistance in both models. **c** Integrative systems biology analysis of proteome and transcriptome data to identify key drivers of resistance. Each node represents a gene or protein; edges represent interaction between genes or proteins. Node color intensity indicates degree of upregulation (red) and downregulation (green) during resistance compared to day 3 responding or untreated samples. **d** Immunoblot analysis of CTSB expression in 786-O xenograft tumor lysates from untreated mice or from mice showing resistance to sunitinib

We have developed a model of human RCC VEGFR TKI resistance in murine xenografts. These VHL-deficient human RCC xenografts initially respond to VEGFR TKI treatment with reduced tumor growth rates. Eventually, however, these tumors manifest TKI resistance, reinitiating growth despite continued TKI treatment such that long-term tumor stabilization is not achieved^3,13^. To identify candidate protein markers linked to sunitinib resistance in RCC, we performed quantitative proteome profiling on xenograft tumors obtained under three conditions: treatment-naive mice (“untreated”), tumors briefly exposed to sunitinib (“responding” or “Day 3”), and tumors harvested at the time of sunitinib resistance (~Day 30–40). Proteomic profiling was performed on two separate mouse xenograft models of human VHL-deficient RCC, derived from 786-O and A498 cell lines. A total of 422 proteins in 786-O xenografts and 312 proteins in A498 xenografts were identified with at least 1 high confidence ( > 95%) peptide (Fig. 1a). Sunitinib resistance (i.e., resistance signature) was identified by comparing expression of proteins in untreated, TKI-responding, and TKI-resistant tumors in 786-O and A498 cell line RCC xenograft mouse models. Resistance signatures were identified in 786-O (55 proteins) and in A498 RCC xenograft models (31 proteins; Fig. 1b) using fold change-based comparisons among different groups and by implementing self-organizing maps, a structural pattern extraction approach^14,15^. Functional enrichment analysis of TKI resistance-related proteins revealed association with activation of functions related to cell proliferation, progression, cell viability and apoptosis, and migration of endothelial cells (Fig. S1A). Further pathways enrichment analysis depicted significant association of resistance signature with multiple cell proliferation pathways (e.g., “Protein Ubiquitination”, “TWEAK signaling”, “mTOR Signaling”, “autophagy”, “Death Receptor Signaling”) and with immune and inflammation pathways (e.g., “Inflammasome pathway”, “Tumoricidal Function of Hepatic Natural Killer Cells”, “Cytotoxic T Lymphocyte-mediated Apoptosis “, “Acute Phase Response Signaling”) (Fig. S1B). Further comparative analysis of the resistance signatures identified six proteins (CERU, TRFE, EST1, CTSB, IF4B, DBPA) for which altered expression was associated with resistance in both 786-O and A498 RCC xenograft models (Fig. 1b). The heatmap clearly depicts differences in expression of these proteins between “resistant” vs. “day 3” treated, as well as vs. untreated tumors (Fig. 1b).

### Integrative systems biology analysis identified CTSB as a candidate key driver of resistance

Integrative systems biology analysis on transcriptome and proteome data related to sunitinib resistance was performed using an IPA tool. The scale-free network for each of the six resistance-related proteins was generated on the basis of protein-protein, protein-DNA, and protein-RNA interactions. The interactive analysis depicted CTSB and its first-degree neighbors interacting with 33 genes that are also significantly dysregulated in sunitinib resistance-related gene expression data, consistent with dysregulation of a CTSB-controlled module during resistance (Fig. 1c). CTSB emerges from the integrative transcriptome and proteome analysis as the gene product most highly upregulated in the TKI-resistant state in both xenograft models. Immunoblot analysis confirms increased CTSB expression at the time of resistance to therapy (Fig. 1d).

### Stable CTSB knockdown suppresses cell growth of RCC in vitro

**Fig. 2:**
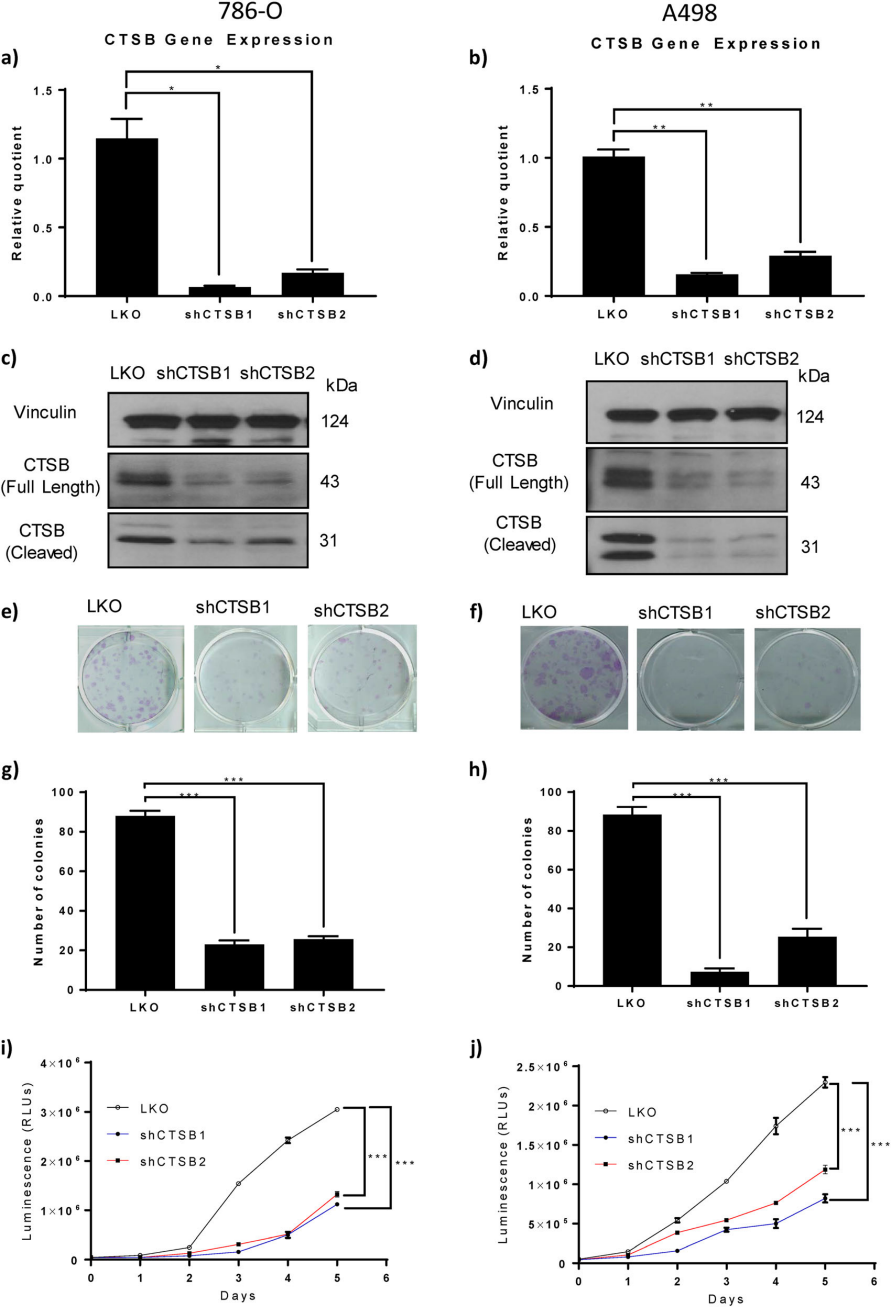
Stable CTSB knockdown in two cell lines suppresses cell growth of RCC in vitro. **a**, **b** CTSB knockdown in two independent clones (shCTSB1 and shCTSB2 vs vector control LKO) reduced RNA levels of CTSB as assayed by real-time PCR. **c**, **d** Reduced levels of full-length and cleaved (active) CTSB protein, as assayed by immunoblot. **e**–**h** CTSB reduction (shCTSB1 and shCTSBs2) in both the 786-O and A498 lines reduced colony formation as compared to LKO. **i**–**j** CTSB down-modulation in 786-O or in A498 cells reduced cell proliferation, as assessed by luminometry

To examine the role of CTSB in regulating RCC cell growth, we suppressed CTSB expression by RNA interference. Two different short hairpin RNA (shRNA) sequences targeting CTSB, in addition to a non-target shRNA control, were used to generate stable cell lines in 786-O and in A498 cells. qRT-PCR (Fig. 2a, b) confirmed suppression of CTSB mRNA levels by each of the two CTSB shRNAs. Similar suppression by both shRNAs was evident in immunoblots (Fig. 2c, d) as reduced levels of both the mature/cleaved CTSB polypeptide (processed, active form) and its full-length precursor protein. To examine whether CTSB knockdown inhibits RCC cell line growth, two CTSB shRNA-expressing cell lines and a vector control cell line (LKO) were derived from parental 786-O and A498 cells and seeded into 96-well plates. Both CTSB knockdown constructs showed significantly inhibited tumor cell colony formation when compared to vector control cell lines (*p* < 0.01) (Fig. 2e–h). CTSB knockdown also suppressed tumor cell proliferation as compared to controls (*P* < 0.01) (Fig. 2i, j). These results were observed in CTSB knockdown cell lines derived from both 786-O and A498 RCC cells.

### Stable overexpression of wild-type CTSB (CTSB^wt/hi^) or CTSB active site mutant (CTSB^N298A^) rescues impaired cell growth in CTSB knockdown cells

**Fig. 3:**
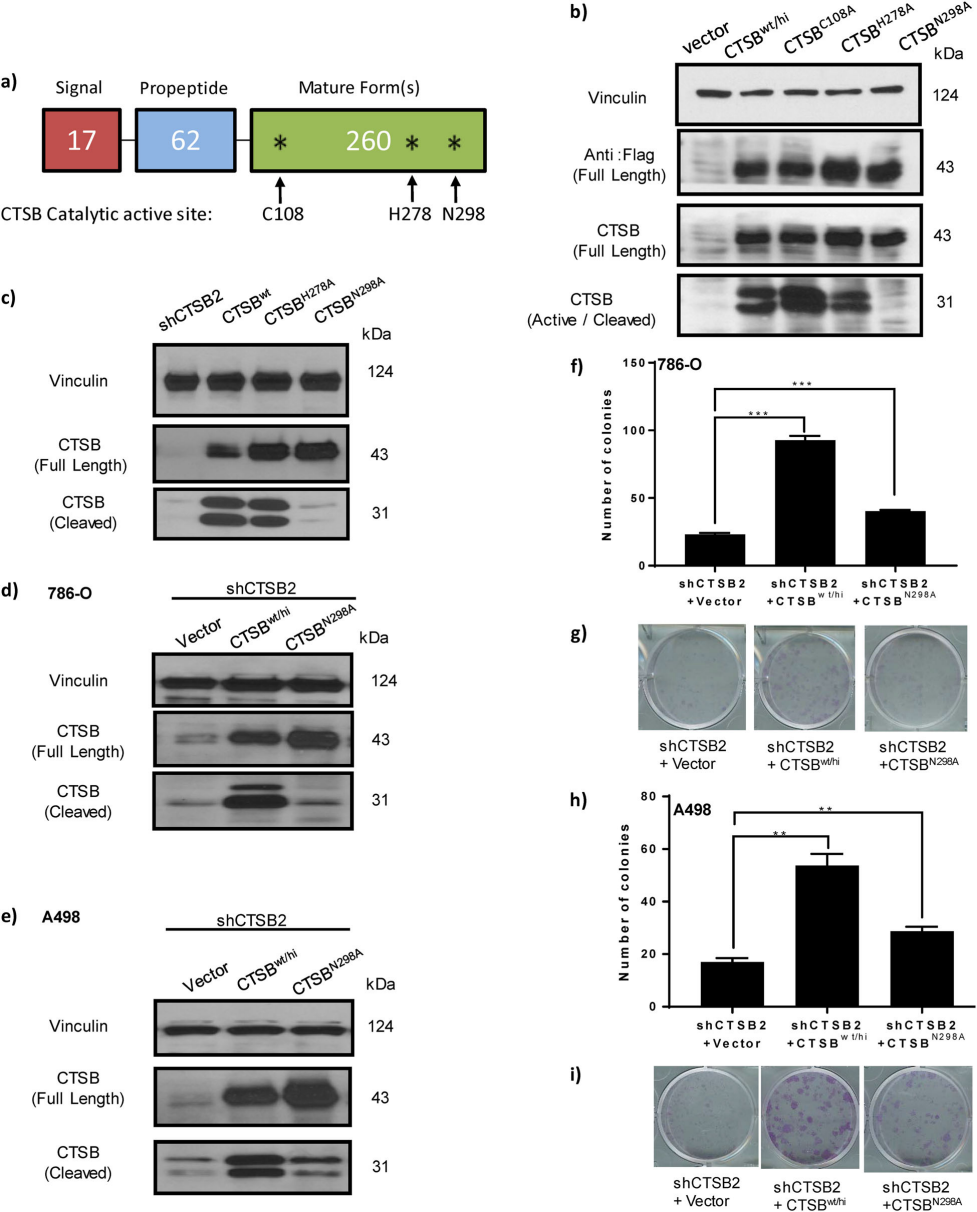
Stable overexpression of wild-type CTSB (CTSB^wt/hi^) but not of an active site CTSB mutant (CTSB^N298A^) rescues impaired cell growth in CTSB knockdown cells. **a** Schematic representation of full-length CTSB shows domains and location of putative enzymatic active sites (asterisk). The mutations generated are indicated with arrows. **b** Immunoblot analysis of CTSB protein levels in five 786-O-derived cell lines: vector control, CTSB stable overexpression of wild-type CTSB (CTSB^wt/hi^), and stable overexpression of three putative active site mutants (CTSB^C108A^, CTSB^H278A^, and CTSB^N298A^). Cleaved (active) CTSB is greatly reduced in the setting of overexpression of the CTSB^N298A^ mutant. **c** CTSB full-length and cleaved forms are down-modulated in the setting of stable shCTSB expression, but only the cleaved form of CTSB is absent in the setting of CTSB^N298A^ overexpression. **d**, **e** Immunoblots show stable overexpression of wild-type CTSB^wt/hi^ or active site CTSB mutant (CTSB^N298A^) in both the 786-O and A498 shCTSB2 knockdown lines. **f**, **g** Colony formation by 786-O lines of three genotypes: shCTSB2 + LKO; shCTSB2 + CTSB^hi^, and shCTSB2 + CTSB^N298A^, demonstrating that in both cells lines, overexpression of wild-type CTSB (CTSB^wt/hi^), but not of active site CTSB mutant (CTSB^N298A^), rescues cell growth in the setting of endogenous CTSB knockdown. **h**–**l** Colony formation by A498 lines of three genotypes: shCTSB2 + LKO; shCTSB2 + CTSB^hi^, and shCTSB2 + CTSB^N298A^

CTSB is biosynthesized on the rough endoplasmic reticulum (RER) as a preproenzyme of 339 amino acids (aa) (Fig. 3a). The N-terminal 17 aa signal peptide directs the protein into the lumen of RER, where the signal peptide is removed, yielding the inactive precursor, procathepsin B. In the acidic environment of lysosomes, procathepsin B can undergo autocatalytic activation as a result of proteolytic cleavage of the propeptide, leading to dissociation of active (mature/cleaved) CTSB. By analogy with other cysteine cathepsin family members, we identified three potential active site residues in CTSB: Cys108, His278, and Asn298. We hypothesized that missense mutation of these residues would disrupt catalytic activation of CTSB (Fig. 3a).

To generate CTSB-Flag inactive mutants, we performed site-directed mutagenesis on each of the three putative active sites and generated cell lines expressing the mutant forms. We next performed western analysis using anti-Flag and two anti-CTSB antibodies, which detected the expression of either full-length (~43 kDa) or processed CTSB (~31 kDa) (Fig. 3b). All three full-length CTSB mutants accumulated to wild-type levels. However, the cleaved mature form of CTSB^N298A^ failed to accumulate, confirming either loss of auto-cleavage activity or gain of instability (Fig. 3b). We next generated stable cell lines and tested CTSB variant expression in 786-O cells. Consistent with results from transient transfections, both proCTSB^H278A^ and proCTSB^N298A^ accumulated to wild-type levels, but the mature, cleaved form of CTSB^N298A^ was greatly reduced in abundance (Fig. 3c). These results suggest that only the mutant Asn298Ala, and not the other point mutations, significantly abrogated activating processing of CTSB (Fig. 3b, c). We next asked whether this mutation affects cell growth-regulatory properties of CTSB. 786-O and A498 CTSB knockdown cells were reconstituted with stable expression of wild-type CTSB (CTSB^wt/hi^) or mutant CTSB^N298A^ (Fig. 3d, e). The 786-O vector or shCTSB knockdown cells stably re-expressing CTSB and CTSB^N298A^ were seeded on plastic plates for proliferation assays. Indeed, cell proliferation was rescued by overexpression in 786-O knockdown cells of wild-type CTSB, but not by overexpression of its inactive mutant (Fig. 3f, g). Similar results were seen in A498 RCC cell lines (Fig. 3h, i). These findings together confirm the functional role of CTSB and further confirm the importance of putative active site residue Asn298, as critical to in vitro cellular function of CTSB.

### Stable CTSB knockdown prevents tumor formation

To evaluate the impact of CTSB on tumor growth in vivo, we generated tumor xenografts with our 786-O CTSB knockdown cell lines. The shCTSB1 xenograft model yielded tumor formation in only one mouse from the ten mice implanted. In contrast, 17 of 20 (85%) mice injected with 786-O shControl cells developed tumors. Implantation of the 786-O shCTSB2 cell line, which expressed intermediate CTSB levels, resulted in tumors in 8 of 15 (53%) injected mice.

### Stable CTSB knockdown suppresses tumor growth of RCC xenografts

**Fig. 4:**
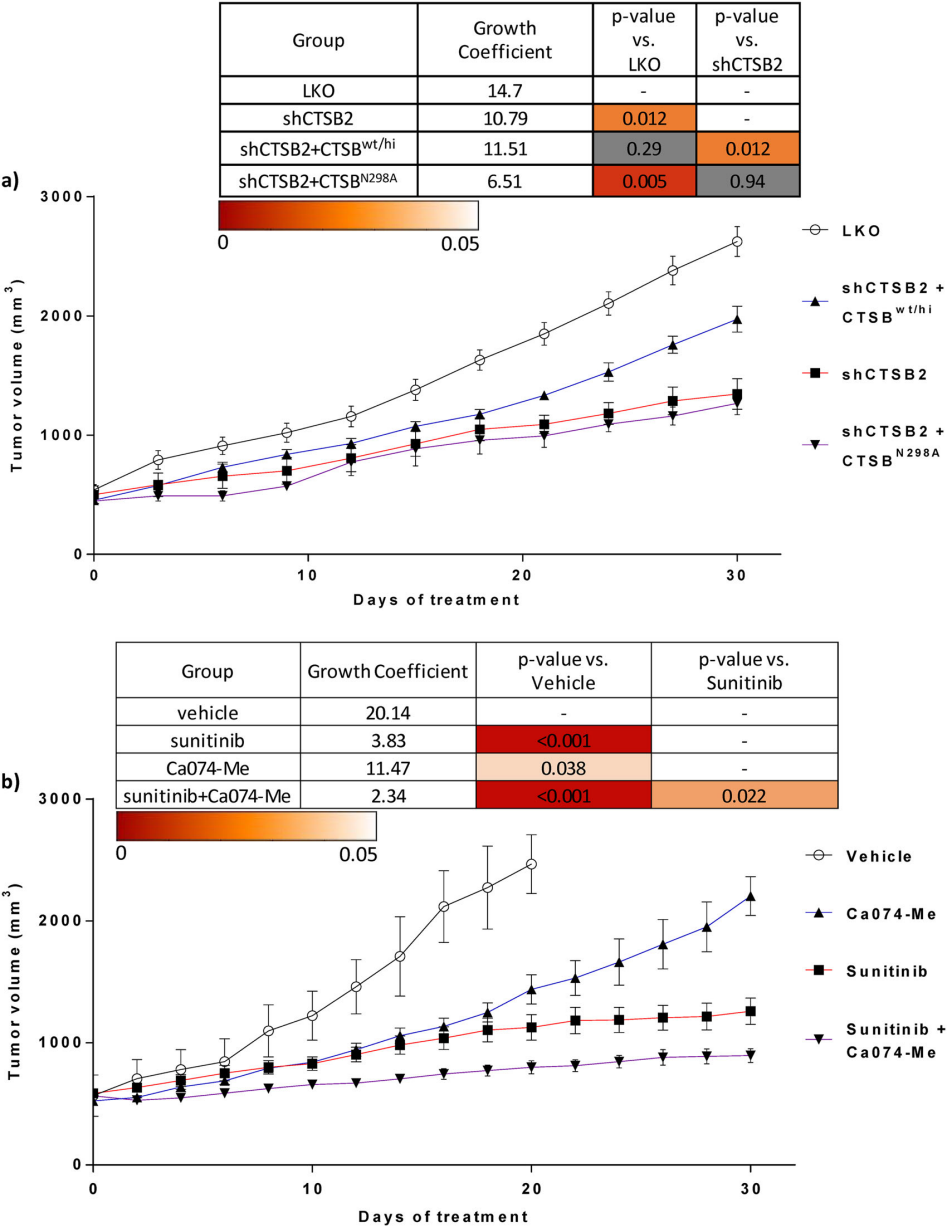
Stable CTSB knockdown suppresses tumor growth of RCC xenografts—an effect rescued by wild type but not mutant CTSB. Pharmacological inhibition of CTSB in combination with sunitinib is superior to sunitinib monotherapy. Tumor growth curves and tumor growth coefficients and *P*-values are shown in tables above curves, with table cells colored as per linear scale color gradient to represent *P*-values. Non-significant values are displayed with gray shading. **a** tumor growth in the 786-O model of LKO vs shCTSB2. shCTSB2 + CTSB^wt/hi^ xenografts exhibited partial rescue from the growth-inhibitory effects of shCTSB, whereas shCTSB + mutant CTSB^N298A^ xenografts failed to exhibit rescue of the growth suppression seen with shCTSB. **b** Effects of pharmacologic CTSB inhibition in the A498 tumor model. Tumor growth was reduced by single agent Ca074-Me but to a lesser extent than with sunitinib alone. The combination of Ca074-Me and sunitinib showed stronger growth inhibition than did either single agent

Tumor growth in mice was assessed over 30 days post-tumor cell implantation. Tumors generated from stable shCTSB2 knockdown cells showed significantly reduced growth coefficients compared to those of vector control (LKO) tumors (*P* = 0.012) (Fig. 4a). The harvested shCTSB2 tumors exhibited significantly reduced CTSB RNA (Fig. S2A) and protein (Fig. S2B). To further examine the functional consequences of CTSB in vivo, the shCTSB2 cell line was transduced to overexpress either wild-type CTSB (CTSB^wt/hi^) or active site mutant CTSB^N298A^. Overexpression of wild-type CTSB in the shCTSB2 line mitigated the reduction in tumor growth seen in shCTSB2 tumors, such that tumor growth was not significantly different from that of the vector control group (*P* = 0.29). In contrast, overexpression of CTSB^N298A^ in the shCTSB2 line failed to rescue the growth-inhibitory effects of CTSB knockdown (*P* = 0.94 for shCTSB2 tumor growth vs shCTSB2 + CTSB^N298A^ tumor growth) (Fig. 4a).

### Pharmacologic inhibition of CTSB suppresses tumor growth of RCC xenografts

Next we tested whether combined treatment with CTSB inhibitor, Ca074-Me, and VEGFR TKI, sunitinib, could provide incremental benefit compared to sunitinib monotherapy. A498 cell RCC xenografts were generated. Mice were randomized into four treatment arms: (1) vehicle; (2) Ca074-Me; (3) sunitinib; and (4) sunitinib plus Ca074-Me as combination therapy. Treatment with either sunitinib (Su) alone or Ca074-Me alone slowed A498 xenograft growth significantly compared to vehicle treatment (vehicle vs sunitinib: *P* < 0.001; vehicle vs Ca074-Me: *P* = 0.038) (Fig. 4b). Combination treatment with Ca074-Me and sunitinib led to a further reduction in growth coefficient compared to sunitinib monotherapy (*P* = 0.022) (Fig. 4b).

### CTSB overexpression leads to faster tumor growth and desensitizes tumors to sunitinib activity in RCC xenografts

**Fig. 5:**
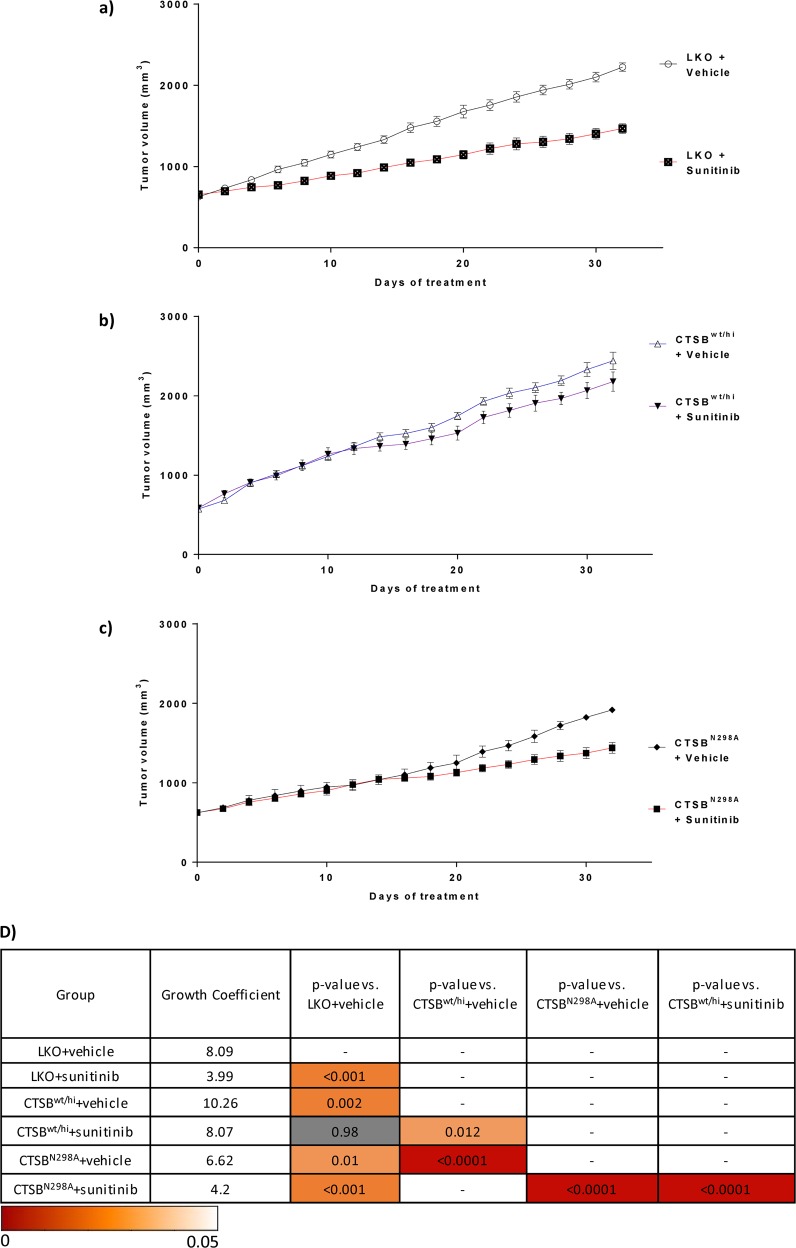
CTSB overexpression desensitizes tumors to sunitinib. Growth curves from the 786-O tumor model. **a**–**c** Mice bearing tumors derived from the vector control (LKO) line, the CTSB^hi^ cell line, or the mutant CTSB^N298A^ line were treated with either sunitinib or vehicle control. Sunitinib treatment reduced tumor growth in LKO and CTSB^N298A^ to similar extents. CTSB^wt/hi^ + sunitinib xenografts grew faster than either LKO + sunitinib xenografts or CTSB^N298A^ + sunitinib xenografts. **d** *P*-values for all relevant comparisons are shown. Growth coefficients are also shown. Table cells colored as per linear scale color gradient to represent *P*-values. Non-significant values are displayed with gray shading

As a corollary to the CTSB knockdown-associated reduction in tumor growth, we assessed the impact of CTSB overexpression on RCC 786-O xenografts. Wild-type CTSB-overexpressing (CTSB^wt/hi^) and uncleaved active site mutant CTSB-overexpressing (CTSB^N298A^) RCC cell lines were generated. CTSB^wt/hi^, CTSB^N298A^, and vector control (LKO) cell lines were used to generate tumor xenografts. Cells were injected in mice, and tumor growth was assessed over 30 days. In the absence of sunitinib treatment, vehicle-treated CTSB^wt/hi^ tumors exhibited significantly higher growth coefficients compared to vehicle-treated LKO tumors (*P* = 0.002; Fig. 5d). Consistent with previous findings, sunitinib-treated LKO tumors also exhibited a significantly lower tumor growth coefficient compared to vehicle-treated LKO tumors (*P* < 0.001; Fig. 5a).

We next hypothesized that in RCC cell lines, overexpression of wild-type CTSB (CTSB^wt/hi^) but not CTSB mutant N298A (CTSB^N298A^) would decrease sunitinib responsiveness. Sunitinib-treated CTSB^N298A^ cell-derived tumors showed significantly lower tumor growth coefficients than vehicle-treated CTSB^N298A^ tumors (*P* < 0.0001; Fig. 5c). Remarkably, growth coefficients for sunitinib-treated CTSB^N298A^ tumors and sunitinib-treated LKO tumors were similar (*P* = 0.73). Among the three xenograft models LKO, CTSB^wt/hi^, and CTSB^N298A^, sunitinib treatment was least effective in retarding tumor growth in the setting of wild-type CTSB overexpression (Fig. 5b). Respective growth coefficients for the sunitinib-treated xenograft models LKO, CTSB^N298A^, and CTSB^wt/hi^ were 3.99, 4.2, and 8.07. These data together show that CTSB^wt/hi^ overexpression decreases responsiveness to sunitinib, whereas CTSB^N298A^ responds to sunitinib treatment in a manner similar to that of LKO controls.

### Genome-wide expression profiling identified ALDH1 as a CTSB target required for tumorigenicity of RCC

**Fig. 6:**
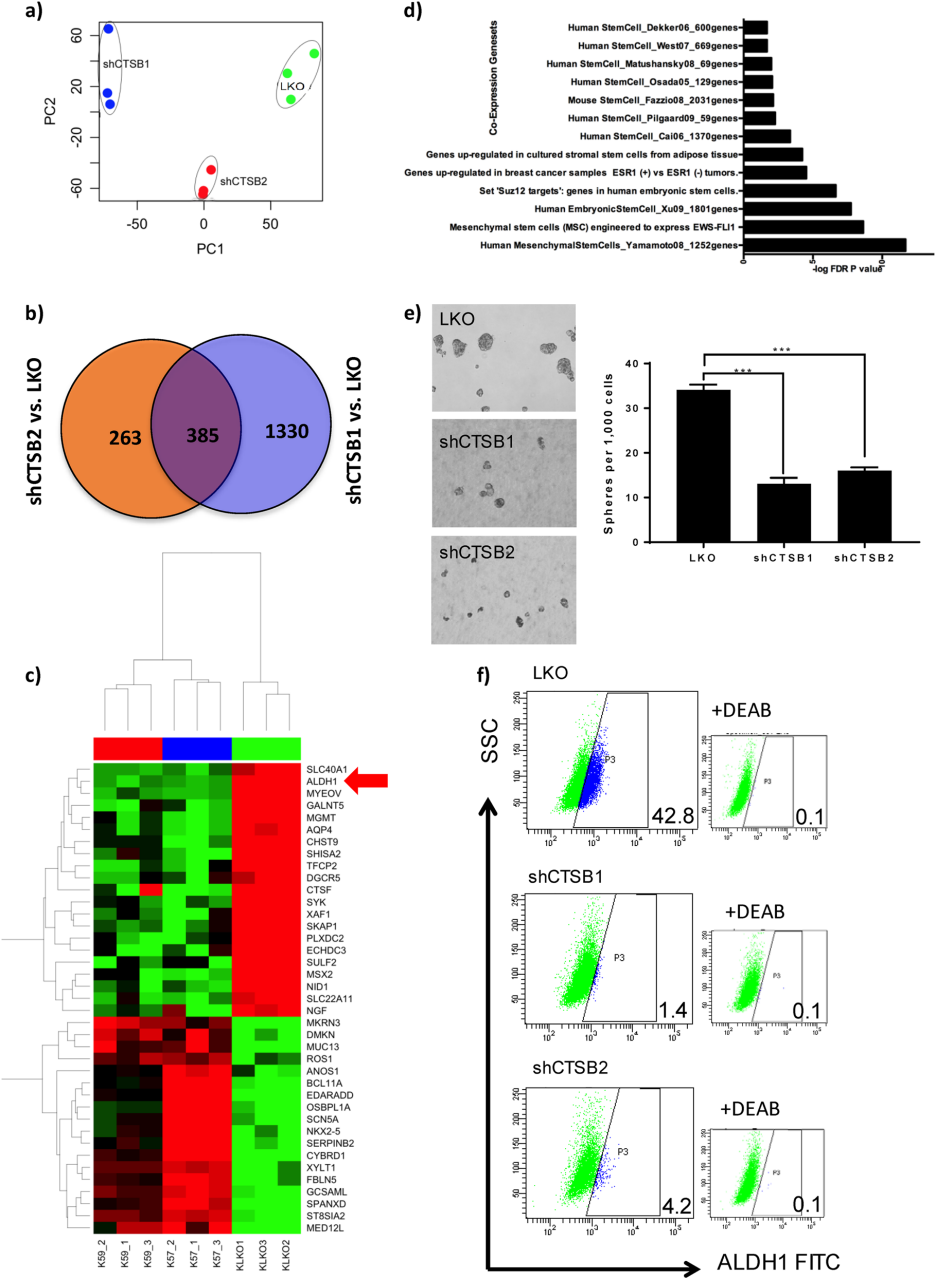
Genome-wide expression profiling identified ALDH1 as a CTSB target requited for tumorigenicity of RCC. **a** Principal Component Analysis of RNASeq data from Control and CTSB-knockdown cells. The first component with highest variance is on the *x*-axis separating control (i.e., Vehicle) from CTSB- knockdown samples (i.e., shCTSB1, shCTSB2), **b** Venn Diagram analysis to identify genes consistently altered by CTSB- knockdown using different siRNAs (i.e., shCTSB1, shCTSB2). The analysis identified 385 genes significantly altered (Absolute Fold Change ≥2 and FDR < 5%) by CTSB- knockdown. **c** Heatmap of genes most highly altered in expression due to CTSB-knockdown. Columns represent samples and rows represent genes. Gene expression is shown in pseudocolor with red denoting increase and green decrease in gene expression. ALDH1 is one of the genes most highly altered by CTSB knockdown. **d** Enrichment analysis of CTSB knockdown-altered genes in coexpression based gene sets, with significance estimated by Fisher’s Exact Test *p*-value, depicted as –log10 (*p*-value) on the primary *x*-axis, **e** CTSB KD reduced sphere-forming activity. Results shown are mean + /− SEM, *n* = 3. **P < 0.001. **f** CTSB knockdown reduced ALDH1 activity. This decreased activity is manifest as a shift in the ALDH1 + cell population to the left, similar to the shift seen in the setting of staining with DEAB, an inhibitor of ALDH enzyme activity

Having demonstrated the fundamental role of CTSB in tumor growth, we next attempted to elucidate the underlying molecular mechanisms. We analyzed the effects of CTSB knockdown on gene expression using RNA Sequencing. Unsupervised principal component analysis demonstrated that samples segregated by CTSB status (e.g., knockdown vs. vector control) along principal component 1 (PC1). CTSB_shRNA1, CTSB_shRNA2, and vector control transcriptomes clustered separately (Fig. 6a), indicating their different transcriptome landscapes. Supervised analysis of CTSB knockdown vs. control cells identified 385 genes significantly dysregulated (FDR < 5% and Fold Change 2) in both shRNA experiments (Fig. 6b). Of 385 genes similarly changed in both CTSB knockdown experiments, 136 were downregulated and 249 were upregulated (Table S1). Among the genes downregulated in the setting of CTSB knockdown were many linked to CSC phenotypes, including ALDH1 (ALDH1A1), SLC40A1 (ferroportin), and PLXDC2 (Fig. 6c).

To further investigate possible mechanisms underlying CTSB knockdown-induced alterations, we performed pathways enrichment analysis to examine relevant gene ontology (GO) categories and coexpression gene sets. GO category analysis of CTSB knockdown-downregulated genes highlighted (*P* < 0.05) immune and inflammatory responses, cell differentiation, and metabolic processes (Fig. S3A). Pathways analysis of differentially expressed genes identified multiple metabolic pathways (*P* < 0.05), including “Chondroitin Sulfate Biosynthesis”, “Eicosanoid Signaling”, “Melatonin Degradation I”, “L-cysteine Degradation II”, “Tight Junction Signaling”, “CDK5 Signaling”, and “Atherosclerosis Signaling” (Fig. S4B). Further geneset enrichment analysis of differentially expressed genes highlighted enrichment of CTSB knockdown-altered genes in Stem Cell-related gene sets, suggesting a possible role of CTSB in driving or maintaining stemness (Fig. 6d). We therefore tested the role of CTSB in regulating tumor-initiating stem cells by assaying tumor cell sphere formation. Stable CTSB silencing with two CTSB shRNAs in 786-O cells reduced both size and number of spheres (Fig. 6e). As ALDH1 is a known marker for CSCs, we measured the effect of CTSB knockdown on ALDH1 enzymatic activity. CTSB knockdown reduced the proportion of ALDH1^+^ stem cells. Importantly, CTSB effects on stem cell growth were dependent on CTSB catalytic activity, as the CTSB mutant N298A failed to rescue ALDH1 expression in CTSB knockdown cells (Fig. 6f). Furthermore, CTSB knockdown in the 786-O shCTSB1 and shCTSB2 cell lines led to reduced expression of proteins related to the stem cell phenotype including, LIN28A, c-Myc, KFL4, Oct-4A, Sox2, ALDH1, and CTSB itself, and reduced expression of mRNAs encoding ALDH1, CD44, CXCR4, Nanog, Oct4, ALDH1, and VEGF (Fig. S4). Thus, ALDH1 is a strong candidate mediator of CTSB’s regulation of RCC tumorigenicity, possibly by impacting CSC phenotypes.

### The transcription factor STAT3 is an upstream regulator of CTSB

**Fig. 7:**
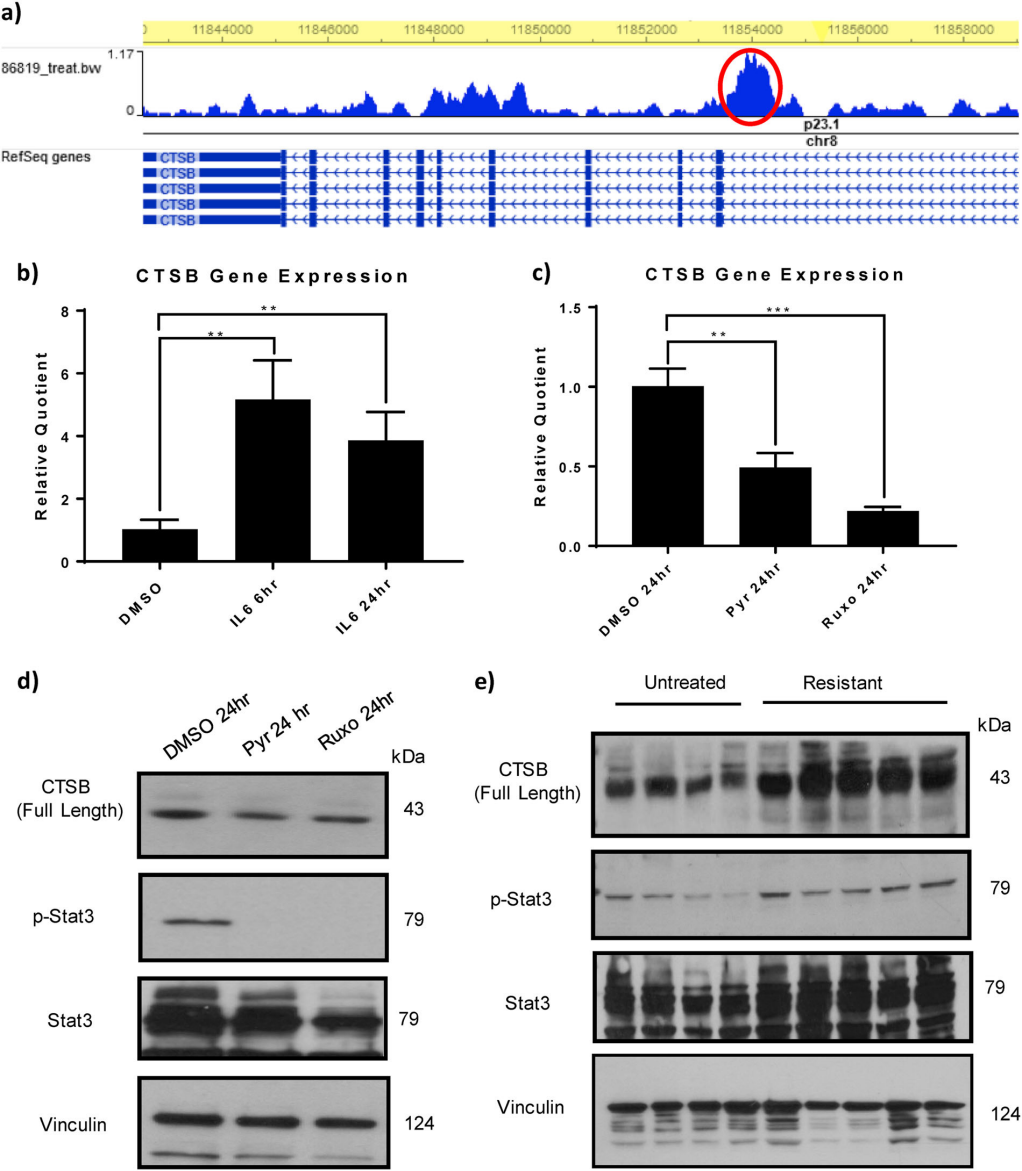
STAT3 regulates CTSB expression in vitro and in sunitinib-treated resistant tumors. **a** STAT3 binds to a potential regulatory region of *CTSB*. ChIP-seq analysis shows several peaks of STAT3 binding, including a major peak (in red) in a potential intronic regulatory region. STAT3 binding was visualized using the Washington University Epigenome Browser (epigenomegateway.wustl.edu/). **b** IL-6 upregulates CTSB mRNA expression in 786-O cells in vitro. **c** CTSB mRNA expression is inhibited by the STAT3 transcriptional inhibitor pyrimethamine and the Jak inhibitor ruxolitinib. **d** CTSB expression and tyrosine phosphorylation of STAT3 are both inhibited by treatment with either pyrimethamine or ruxolitinib. **e** 786-O tumor xenograft lysates from mice confirm the correlation between CTSB and STAT3 activation by immunoblot

To study the upstream and downstream mechanisms of the CTSB pathway in RCC we first assessed the CTSB promoter and identified binding sites for STAT3^16^. To address the physiological relevance of this finding, we treated RCC cells in vitro with IL-6, a tumor microenvironment known to activate STAT3. We found that IL-6 increased CTSB expression at 6 and 24 h following treatment (Fig. 7b). To determine if the expression of CTSB was dependent on STAT3 transcriptional activity, we treated cells with either of two STAT3 inhibitors, the transcriptional inhibitor pyrimethamine^17^, or the upstream JAK kinase inhibitor, ruxolitinib. Both treatments decreased expression of CTSB (Fig. 7c, d). To further assess whether STAT3 could be regulating *CTSB* expression in sunitinib-treated murine tumors, we measured levels of the transcriptionally active, tyrosine-phosphorylated form of STAT3. Sunitinib-resistant tumors showed elevated CTSB expression, as predicted, in correlation with increased phosphorylated STAT3 (Fig. 7e). Moreover, sunitinib had no direct effect on tumor cell CTSB expression in vitro (Fig. S5). These data show that the STAT3 pathway regulates CTSB expression in vivo and in vitro.

### CTSB association with survival in a pan-cancer dataset

**Fig. 8:**
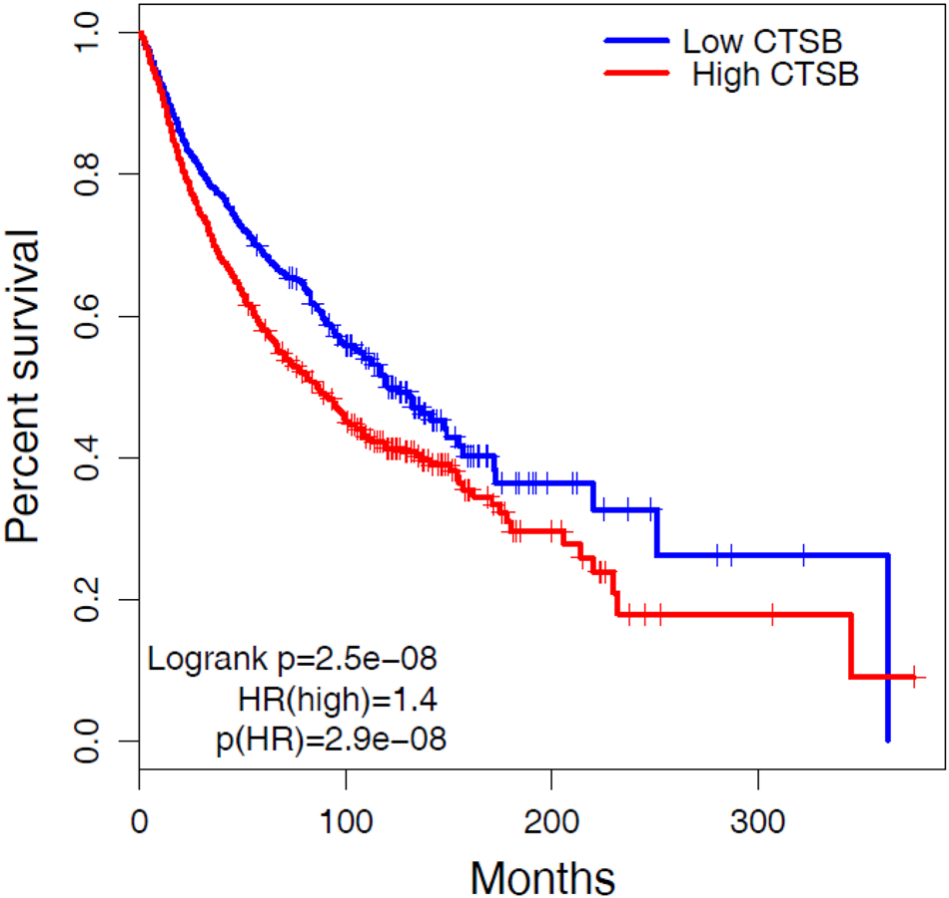
CTSB association with survival in the TCGA pan-cancer dataset. To explore the association of CTSB with survival, we generated from TCGA data Cox proportional hazard and Kaplan–Meier models of survival analysis. Data was partitioned into CTSB High (Top 25%) and Low (Bottom 25%) groups based on high and low quartiles of CTSB expression. The Kaplan–Meier analysis revealed that higher CTSB expression (red curve) is significantly associated with reduced survival (*P* ≤ 0.05)

To explore association of CTSB expression with survival across all cancer types, we performed survival analysis using the TCGA database. The TCGA database contains > 30,000 clinically and genomically characterized tumors from major cancer types, including RCC. Survival analysis was performed on tumor samples in the upper quartile (high CTSB) and lower quartile (low CTSB) of CTSB expression. Analysis of the 2,376 samples in the CTSB high and low groups revealed that higher CTSB expression of CTSB is significantly associated with reduced survival (*P* ≤ .05) (Fig. 8). The clinical information from the TCGA database allowed construction of a Cox proportional hazards model predicting a patient’s survival according to CTSB expression. Higher CTSB expression was significantly associated with poor survival in the pan-cancer dataset (HR > 1.4). These results indicate that elevated CTSB expression may drive RCC growth or TKI resistance, leading to poor clinical outcomes by increasing tumor stemness phenotype.

## Discussion

RCC is a tumor-type sensitive to VEGFR TKI therapy, likely a result of the Von Hippel-Lindau (VHL) gene loss that commonly characterizes these tumors. Accordingly, treatment with VEGFR TKIs such as sunitinib has become established therapy for metastatic RCC, and can lead to periods of tumor stability. However, all patients receiving TKI monotherapy eventually develop resistance. As a consequence, novel therapies and drug targets are being actively sought to overcome TKI resistance. Several pathways have been implicated in the ability of RCC to resist or adapt to anti-angiogenic therapy. Increased proangiogenic pathway activity, including ALK-1, IL8, Notch/Dll4, Ang2, and FGF, as well decreased anti-angiogenic factors, both support angiogenesis in the setting of VEGFR inhibition^3,18–23^. Anti-angiogenic therapy can induce tumor hypoxia, and pathways upregulated in the setting of tumor hypoxia, such as sphingosine phosphate and c-Met/HGF signaling, also contribute to TKI resistance^24–26^. Indeed, growing interest in developing new therapeutic combinations to improve clinical responses has led to introduction of Cabozantanib, which combines inhibition of VEGF and Met to produce outcomes better than obtained with VEGF inhibition alone^27^. Additional resistance mechanisms include accumulation of myeloid derived tumor suppressor cells (MDSCs) and epithelial-to-mesenchymal transition (EMT)^28–30^. Lysosomal sequestration has also been reported as a potential resistance mechanism to sunitinib^31,32^. Most recently, combined inhibition of the VEGF and PD-L1 pathways has promised to offer improved clinical outcomes for patients with metastatic RCC^33,34^.

To our knowledge, only one prior report has investigated a role for CTSB in RCC, showing in vitro lysosomal sequestration and inactivation of sunitinib, leading to further lysosomal acidification and a reduction in CTSB levels. Our data suggest that CTSB can show a subsequent compensatory increase in expression during continued sunitinib treatment, and we propose this response could drive sunitinib resistance. Our demonstration of slowed tumor growth in the presence of CTSB knockdown implicates CTSB not only in VEGFR TKI resistance, but also as a potential novel target in treatment of RCC.

In this study, we used proteomic and genomic approaches, along with systems biology techniques, to show the key role that CTSB plays in RCC tumor growth and resistance to TKI therapy. We identified CTSB in both proteome and transcriptome analyses as one of the most highly upregulated gene products in TKI-resistant tumors derived from two independent mouse xenograft models of VHL-deficient RCC. We also demonstrated the functional relevance of CTSB for in vitro and in vivo suppression of RCC growth. Furthermore, we showed that overexpression of CTSB (CTSB^wt/hi^) but not of an active site mutant CTSB (CTSB^N298A^) abolished the therapeutic inhibitory effect of TKI on tumor growth. These data depict CTSB as a key regulator of the TKI resistance phenotype.

CTSB down-modulation also improved the response to VEGFR TKI therapy in vivo. We found ALDH1 as one of the most highly altered CTSB targets, suggesting a path for future investigation of the mechanism by which CTSB affects stem cell activity. We have identified a novel CTSB residue crucial to the active site-mediated auto-cleavage required for proenzyme activation. We showed that mutation of this site inactivates CTSB, information that could support further development of CTSB inhibitors. To our knowledge, there are currently no CTSB inhibitors in clinical trials. Our results open a pathway for potential development of novel CTSB inhibitors and alternative approaches to combat RCC resistance to TKI treatment.

The possible link between CTSB and cancer was first postulated many years ago^35,36^. Several clinical studies since then have shown correlations between CTSB expression and cancer progression and clinical outcomes^37^. Both CTSB mRNA and full-length CTSB polypeptide are more highly expressed in malignant tumors than in benign tumors or normal^38,39^. However, mechanisms and pathways related to CTSB expression remain to be elucidated. Previous data suggest roles for CTSB in tumor invasion and metastasis^11,39–42^. These functions have been attributed to alterations in intracellular trafficking of CTSB frequently observed in malignant tumors, in addition to the ability of CTSB to degrade ECM proteins such as laminin, fibronectin, and collagen^43^.

CTSB has more recently been implicated in stem cell maintenance, as demonstrated in glioma-initiating cells^44^. The involvement of tumor-initiating or CSCs in drug resistance and metastasis has led to design of many therapies intended to target these cells specifically, in the hope of preventing oncologic relapse. CTSB may provide a promising target for such novel cancer therapies. Our study showed that CTSB knockdown in two RCC cell lines reduced tumorigenesis, suggesting the possibility that CTSB functions in maintenance of RCC stem cell-like characteristics. We further showed that CTSB supports CSCs and that inhibition of CTSB can suppress this cell population. Stem cells have been shown to be important in RCC, and RCC stem cell markers described to date include CD133, CD44, CXCR4, CD105, and Spalt-Like Transcription Factor 4 (SALL4)^8,45–48^. High ALDH1 expression in RCC confers stem cell properties such as sphere-forming capacity in vitro^49^. ALDH1 gene expression has also been correlated with tumor grade but not tumor stage in patients with RCC^50^. Overall pooled analysis suggests that high expression of CSC markers (including CD133, CD44, CXCR4, and CD105) predicts poor overall survival, cancer-specific survival, disease-free survival, and progression-free survival^50^. However, the molecular mechanism connecting ALDH1 and stem cells in RCC remains to be fully explored. We have shown that CTSB knockdown slows tumor growth and improves response to VEGFR TKI therapy by mechanisms consistent with an effect on ALDH1-expressing CSCs. We have also shown that STAT3 can regulate CTSB and that perhaps this is the mechanism for induction in vivo. Thus, our data are consistent with the hypothesis that in vivo, sunitinib treatment affects the tumors by causing stress (metabolic, hypoxic etc) and that induces the STAT3 pathway. This leads to production of CTSB which can promote tumor growth.

A strength of our study is the use of an active site mutant of CTSB to show specificity of the CTSB effect. The overexpressed mutant polypeptide fails to undergo maturational cleavage to the active CTSB and fails to rescue the impaired growth phenotype of CTSB knockdown, in contrast to the successful rescue by overexpression of wild-type CTSB. To our knowledge this is the first report of this functional CTSB mutation. Another study strength is our demonstration, through TCGA data mining, that patients with high tumoral CTSB expression have worse clinical outcomes than patients with low tumoral CTSB expression. A limitation of the human study is its restriction to primary tumors, whereas CTSB induction by VEGF TKI treatment of advanced RCC may prove more clinically relevant. Unfortunately, pre- and post-VEGF TKI metastatic tumor tissue is not readily available. Another limitation of our study is our reliance on human RCC xenografts in immune-deficient mice. Tests of our hypotheses in an immune-competent model of RCC will be important when a standard model becomes available.

In conclusion, this study provides a rationale for combined inhibition of VEGF and CTSB pathways as a novel therapeutic strategy for patients with metastatic RCC.

## Materials and methods

### Plasmids

The CTSB cDNA was a gift from Hyeryun Choe (Addgene plasmid # 11249) and subcloned to pRK5-Flag or pLenti6/V5-GW/LazC vector^51^. The CTSB mutants with C-terminal Flag were generated by site-directed mutagenesis in which the Cys108, His278 or Asn298 residues were each replaced by an Ala residue, and subcloned into the vector pLenti6/V5-GW/lacZ. shRNA lentiviral constructs targeting human CTSB (shCTSB1: TRCN0000003657 and shCTSB2: TRCN000003659) were from Sigma (St. Louis, MO).

### Cell culture and transfection

Human RCC lines 786-O and A498 were obtained from American Type Culture Collection and maintained in RPMI 1640 or MEM medium, respectively, supplemented with 10% fetal bovine serum (FBS), 100 U/ml penicillin, and 100 mg/ml streptomycin (Gibco/Life Technologies). Cell cultures were maintained at 37 ^o^C under 5% CO_2_. Lipofectamine 2000 (Invitrogen, Grand Island, NY) and shRNA were diluted in Opti-MEM medium (Gibco) and combined at the ratio of 3:1 for transfection per manufacturer’s recommendation. Where indicated, cells were treated with IL-6 (PeproTech; 10 ng/ml), pyrimethamine (Sigma; 10 μM), or ruxolitinib (Shanghai Haoyuan Chemexpress; 10 μM).

### Lentivirus production

CTSB and control shRNA lentiviral constructs were from Sigma, and viral particles were assembled per manufacturer’s recommendations (Invitrogen). pLKO vectors were transfected into HEK293 cells along with the two packaging constructs, and supernatants containing lentiviruses were collected and concentrated by ultracentrifugation. 786-O and A498 cells were infected with lentiviruses supplemented with 8 μg/ml polybrene, and shRNA-expressing cells were selected in 2 μg/ml puromycin. pLKO vector served as control.

### Establishment of stable cell lines

Lentivirus produced in HEK293 cells was used for infection, as described previously^51^. To overexpress CTSB and its mutant constructs in 768-0 and A498 cells, cells were infected with lentiviruses encoding CTSB, its mutants, or a control construct. Infected cells were selected in 5 µg/ml blasticidin. Stable cell clones were checked for protein expression by immunoblot to confirm expected protein expression. Stable cell lines were maintained continuously in culture, passaging every fourth day and seeding at 6 × 10^5^ cells per 10 cm culture dish.

### Immunoblots

Immunoblot analyses were performed as described^52–54^. Relevant proteins were expressed in cells by lentiviral infection, followed by lysis in a buffer containing 50 mM HEPES, pH 7.5, 150 mM NaCl, 100 mM NaF, 1 mM sodium orthovanadate, 10% glycerol, 1% Triton X100, 10 µg/ml aprotinin, 10 µg/ml leupeptin, 50 µg/ml phenylmethylsulfonyl fluoride and 1 mM DTT. Lysates were subjected to immunoblot analysis with the following primary antibodies: rabbit monoclonal anti-CTSB (Abcam, ab125067), mouse monoclonal anti-CTSB (Abcam, ab58802), mouse monoclonal anti-ALDH (BD, 61194), rabbit polyclonal anti-P-STAT3 (Cell Signaling Technology, #9131), rabbit polyclonal ant-STAT3 (Santa Cruz Biotechnology,sc-482), mouse monoclonal anti-α actin (Abcam, ab3280), and rabbit monoclonal anti-Vinculin (Cell Signaling, 13901S).

### Quantitative real-time PCR

Total RNA from cell lines and tumor tissues of xenografts was extracted by TRIzol (Invitrogen). RNA concentration was evaluated by photometric measurement at 260/280 nm. cDNA was transcribed with iSCRIPT cDNA Synthesis Kit (Bio-Rad, Hercules, CA) per manufacturer’s protocol. PCR reactions were performed using 2XSYBR Green qPCR Master Mix (ABgene/ThermoFisher) in the 7600 real-time PCR system (Applied Biosystems, Foster City, CA), with 18S as internal control for samples from cell lines or tumors. Relative mRNA levels were determined by the 2-^ΔΔCT^ formula, and experiments were repeated three times. The paired forward and reverse primers were as follows:

ALDH1 forward 5′-TCCTGGTTATGGGCCTACAG-3′;

ALDH1 reverse 5′-CTGGCCCTGGTGGTAGAATA-3′;

CTSB forward 5′-GTCTTCAGGCCTATGGAGAGC-3′;

CTSB reverse 5′-CATTGGCCAACACCAGCAG-3′;

18sRNA forward 5′-GTAACCCGTTGAACCCCATT -3′;

18sRNA reverse 5′-CCATCCAATCGGTAGTAGGG -3′.

### Cell proliferation, cell viability, and colony formation assays

Cells grown for 1, 2, 3, 4, or 5 days in 96-well plates were assayed for proliferation by CellTiter-Glo® Reagent, per manufacturer’s protocol (Promega, Madison, WI). Relative fold changes in luminescence normalized to respective “0-day” control cells were expressed as “relative proliferation.” Colony formation assays used 200 cells/well in 6–well plates for 7 days, with subsequent staining by 0.5% crystal violet in 30% ethanol, followed by 10 min fixation at room temperature in 3% formaldehyde.

### Flow cytometric analysis

ALDH1 activity was assessed by flow cytometry using the Aldehyde Dehydrogenase Based Cell Detection Kit (Stemcell Technologies, Cambridge, MA) per manufacturer’s recommendation CSC populations were identified as ALDH + in 786-O and A498 cells. Cells were stained with Aldefluor reagent followed by flow cytometric quantitation. Gate selection for ALDH-positive cells was based on diethylaminobenzaldehyde (DEAB)-mediated inhibition of ALDH1.

### Sphere formation assay

Single cell suspensions were plated on ultra-low attachment plates (Corning Biotech, Tewksbury, MA), at 500 cells/ml in 3:1 serum-free DMEM/MammoCult medium (Stemcell Technologies) with 0.8% Methylcellulose (Sigma). After 6–8 days in culture, spheres were collected by centrifugation, dissociated enzymatically (5 min in 1:1 TrypLE/DMEM at 37 ℃), then mechanically by passage through 26 G needles. Single cells were counted and re-plated at 500 cells/ml for subsequent passages. We used Image J to quantitate sphere area.

### Drug formulation and administration

Ca-074Me (APExBIO; Houston, TX) was diluted in TBS or PBS to 2 mg/ml and dosed intraperitoneally at 25 mg/kg, 6 times a week. Sunitinib malate (clinical grade) was resuspended in citrate buffer (pH 2.3) and administered at 30 mg/kg by daily oral gavage, 6 days per week.

### Tumor xenograft studies

For subcutaneous xenograft tumor models, female athymic nude/beige mice (Charles River Laboratories, Wilmington, MA) were housed and maintained in laminar flow cabinets under specific pathogen-free conditions. All experiments were approved by the Institutional Animal Care and Use Committee at Beth Israel Deaconess Medical Center. To establish RCC tumor xenografts, 786-O and A498 tumor cells were injected subcutaneously (10^7^ cells) into the flanks of 6–8 week old nude beige mice (~20 g on average). When tumors reached 12 mm length along the long axis (~500 mm^3^ in volume), mice were randomized into treatment groups (5–8 mice per group). The person performing tumor measurements was different from the person treating the animals so the measurements were performed in a blinded fashion. Our prior studies showed that VEGFR TKI treatment consistently and markedly decreases tumor vasculature and tumor blood flow (measured by perfusion MRI at day 3–10 of therapy). There followed, however, significant recovery of MRI-measured tumor perfusion despite continued treatment, a marker of development of resistance to VEGFR TKI therapy^13^. Treatment was continued until tumors reached 20 mm length along the long axis or until 50 days after treatment initiation.

### Global proteomic analyses

To identify candidate protein-based markers linked to TKI resistance in RCC, we performed quantitative proteome profiling on xenograft tumors obtained from treatment-naive mice (untreated), obtained after brief VEGFR TKI therapy (“responding” or “Day 3”), or obtained at the time of resistance. Quantitative proteomic profiling was performed on two mouse xenograft models of human VHL-deficient RCC (from 786-O and A498 cell lines) by iTRAQ isobaric labeling (AB Sciex, Foster City, CA) allowing simultaneous identification and relative quantitation of thousands of proteins^55^^,^^56^. Raw data was analyzed by ProteinPilot v3.0 software (AB Sciex) using the Paragon algorithm^57^ enabling rapid matching of MS/MS spectra and iTRAQ relative quantitation. Searches were performed against a comprehensive database generated from SwissProt and Refseq protein sequences. Data were normalized for loading error by bias correction and background correction using ProteinPilot. Bias correction corrects for unequal mixing when combining the eight-labeled samples of one experiment by calculating a scaling factor. Proteins identified by at least 1 peptide at 95% confidence were used for further quality control and differential expression analysis. Each protein also achieved quantitative scores on the basis of weighted average peptide scores for each of the eight-iTRAQ tags to calculate relative expression levels. In this experiment, relative protein expression was calculated normalized to an untreated control sample.

### Quality control and unsupervised and supervised analysis of proteomics data

Quality control analysis was performed on the basis of relative expression values of different proteins to identify outliers, using pair-wise correlation plots, boxplots, principal component analysis (PCA), and unsupervised hierarchical clustering. To identify proteins differentially expressed in TKI-resistant xenografts, we performed comparative analysis of Control, Day 3, and Resistant samples. Initially, the seed of differentially expressed proteins was extracted by performing pair-wise comparison (Untreated vs Day 3, Untreated vs Resistant, Day 3 vs Resistant). Proteins with fold change of 1.5 or greater were considered as putatively differentially expressed. Proteins specifically differentially expressed in resistant vs. untreated and vs. day 3 samples were identified by self-organizing map (SOM) analysis^14,15^ on differentially expressed proteins identified from previous analysis, with SOM clustering on relative protein expression values using Pearson correlation coefficient-based distance metrics. Proteins associated with TKI resistance in more than one xenograft model were identified by Venn Diagram analysis.

### Integrated analysis of transcriptome and proteome data

To supplement our previous transcriptome analyses of untreated, responding (Day 3) and resistant xenograft tumors on VEGFR TKI therapy (i.e., sunitinib, sorafenib)^58^, we performed integrated proteome-transcriptome analyses to identify proteins with interactants significantly altered in the corresponding transcriptome samples. We performed enrichment analysis of interactants of 6 proteins associated with resistance in the proteome data, based on the IPA database. Proteins with interactants significantly enriched at the transcriptome level were considered as candidates for key drivers of resistance.

### Pathways and functional enrichment analysis

Ingenuity Pathway Analysis (IPA 8.0; http://www.ingenuity.com) was used to identify pathways and biological functions affected by proteins and genes specifically associated with RCC TKI resistance. IPA calculates a *p*-value (one-tailed Fisher exact test) for each pathway according to the fit of user’s data to the IPA database. Pathways with *p*-values < 0.05 were considered significantly affected.

### CTSB association to survival across major TCGA cancer types

To determine the association of resistance-related proteins with survival in kidney cancer, we performed survival analysis using RNASEQ data from ~10,000 patients in the TCGA database^59^, by examining patterns of individual gene expression in each cancer. The expression data was divided into high and low expression groups on the basis of quartile expression. Survival analysis results were visualized using K-M survival curves with log rank testing. The results were considered significant if log rank test *p*-values were < 0.05.

### Transcriptome profiling on CTSB knockdown samples using RNA sequencing

To understand mechanisms of CTSB in TKI resistance, we also performed transcriptome profiling on control (“Blank”), and CTSB Knockout samples. For each group, sequencing was performed on three biological replicates. Double-stranded cDNA sequencing libraries were generated using the Illumina TruSeq kit per manufacturer’s protocol. High quality libraries were sequenced on an Illumina Nextseq 500. To achieve comprehensive coverage for each sample, we generated ~30–35 million paired end reads. Raw sequencing was pre-processed, quality checked, aligned to human genome (Hg19), and unique numbers of reads counted^60^^,^^61^. The read count-based expression data was normalized using the voom approach that estimates the mean-variance relationship of the log-transformed transcript counts data to generate a precision weight for each expressed transcript^62^. Differentially expressed transcripts were identified from normalized sequencing data using LIMMA linear model microarray analysis software^63^, based on absolute fold change and multiple test-corrected *p*-values. Transcripts were considered significantly differentially expressed if *p*-values were < 0.05 and absolute fold change was > 2.

### Growth coefficient analysis

Analyses were performed using a mixed-effect regression model with terms for group, time, and group-by-time interaction. Each mouse was treated as a random effect, and an auto-regressive error structure was used to allow for correlation of tumor measurements over time with robust variance estimation to allow for departures from the AR-1 correlation model.

### Data analysis and statistics

Statistical analysis of data was performed with GraphPad Prism 5 software (GraphPad Software Inc., La Jolla, CA) or with statistical functions developed using R language. All data are presented as the means ± SD. Significant differences were determined by two-tailed student *t* test or ANOVA test, where *P < 0.05, **P < 0.01, ***P < 0.001.

## Supplementary information

Figure S1

Figure S2

Figure S3

Figure S4

Figure S5

Supplementary legend file.

Table S1

## Data Availability

All raw genomics and proteomics data will be available to the public without restriction. All raw in vitro and in vivo data will be available to the public without restriction.
