# Supplementary figures and images for: Study of Cathepsin B inhibition in VEGFR TKI treated human renal cell carcinoma xenografts
Source: Oncogenesis. 2019 Feb 22;8(3):15. doi: 10.1038/s41389-019-0121-7 (PMC6386754; doi:10.1038/s41389-019-0121-7)

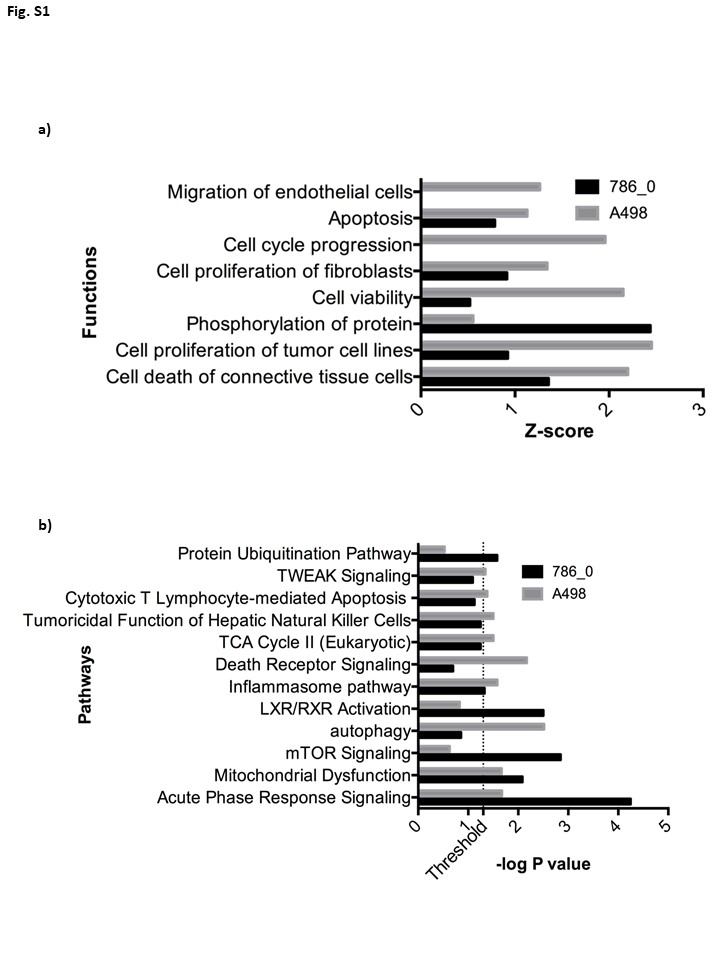

Supplement: Supplementary file 1 — Figure S1 [file 41389_2019_121_MOESM1_ESM.jpg]

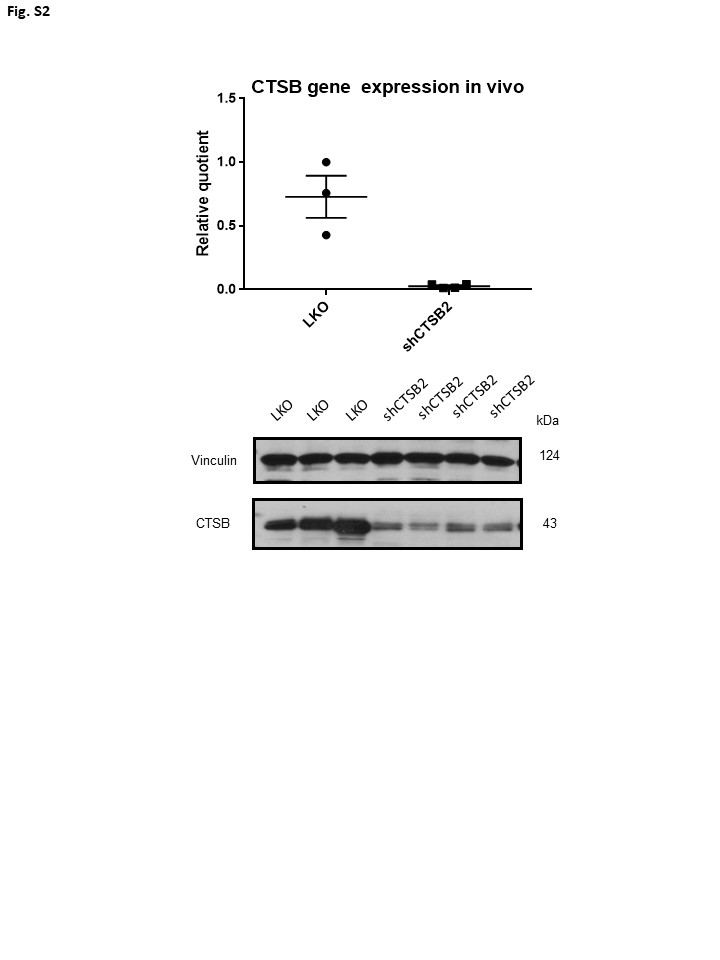

Supplement: Supplementary file 2 — Figure S2 [file 41389_2019_121_MOESM2_ESM.jpg]

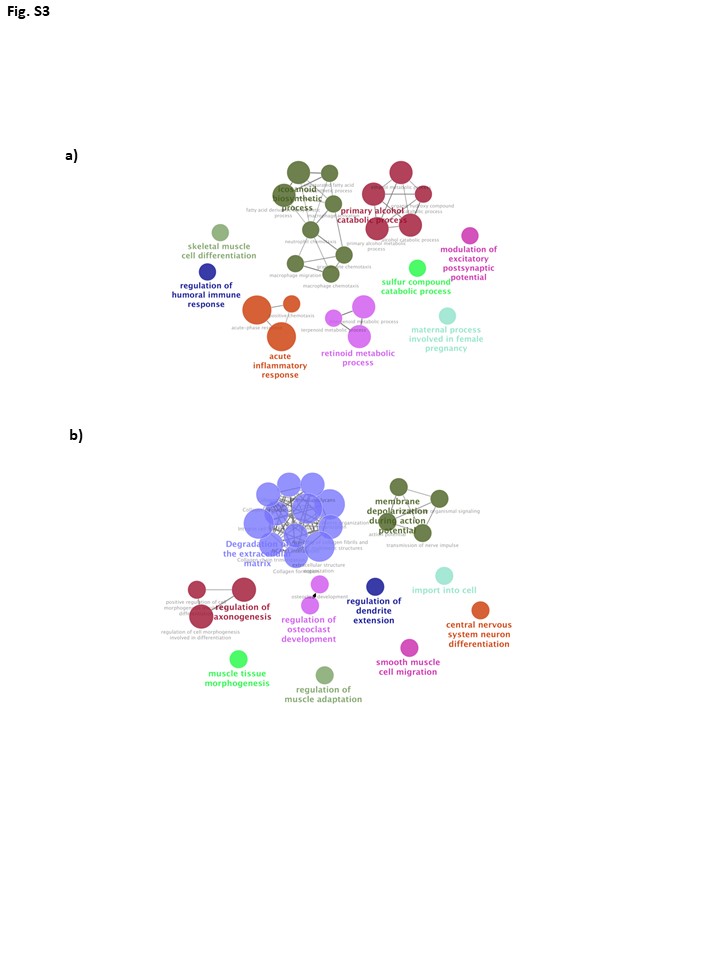

Supplement: Supplementary file 3 — Figure S3 [file 41389_2019_121_MOESM3_ESM.jpg]

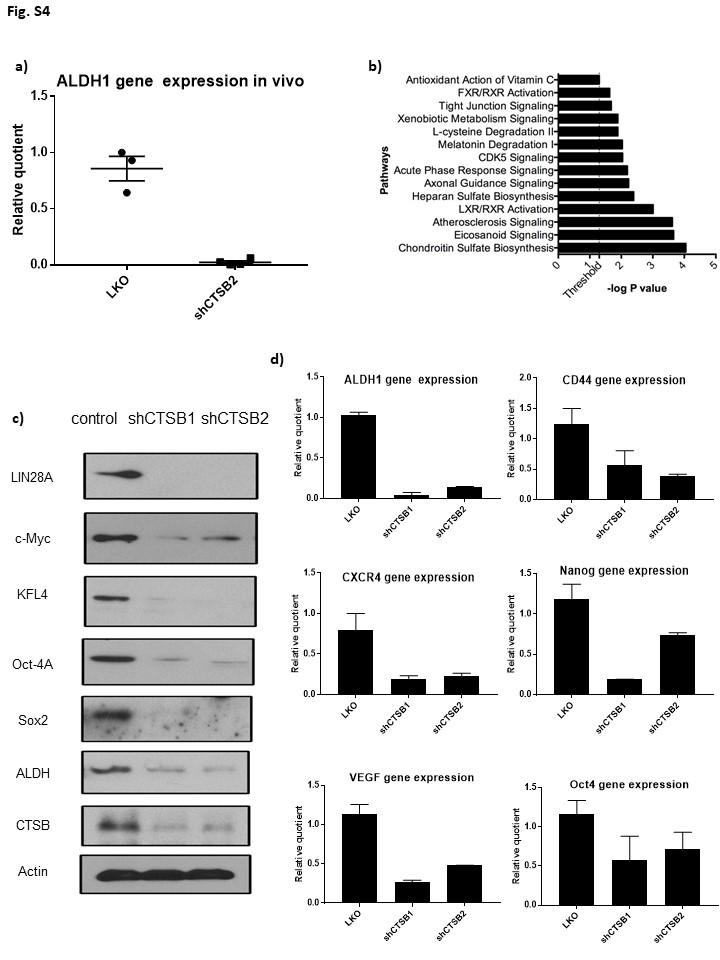

Supplement: Supplementary file 4 — Figure S4 [file 41389_2019_121_MOESM4_ESM.jpg]

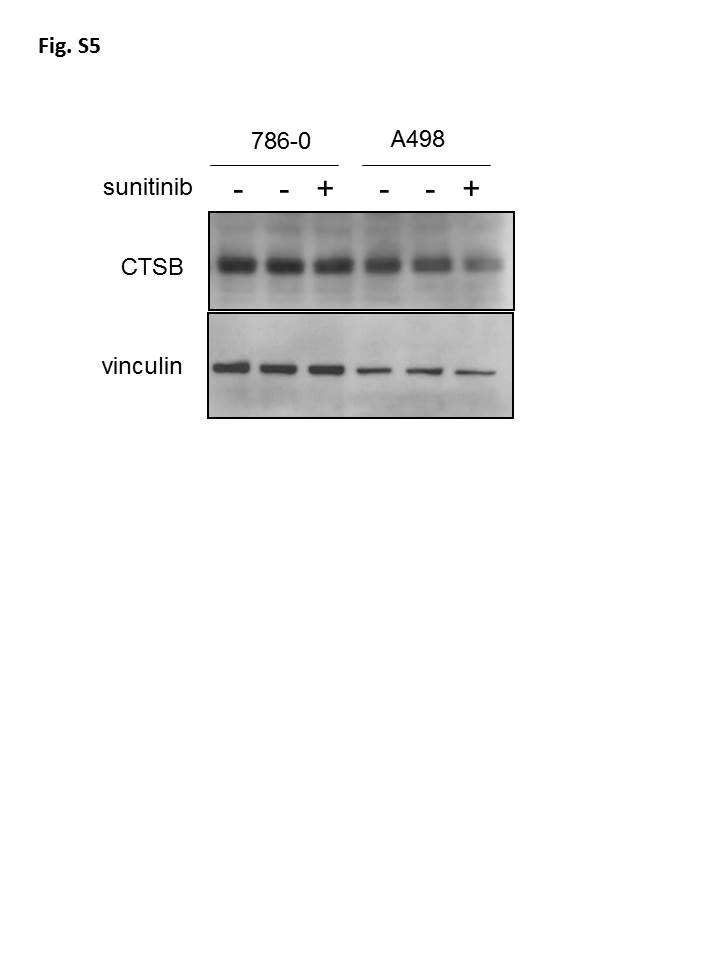

Supplement: Supplementary file 5 — Figure S5 [file 41389_2019_121_MOESM5_ESM.jpg]
