# Supplementary material for: Study of Cathepsin B inhibition in VEGFR TKI treated human renal cell carcinoma xenografts
Source: Oncogenesis. 2019 Feb 22;8(3):15. doi: 10.1038/s41389-019-0121-7 (PMC6386754; doi:10.1038/s41389-019-0121-7)
Supplement: Supplementary file 6 — Supplementary legend file. [file 41389_2019_121_MOESM6_ESM.docx]

**Table S1:** **List of genes consistently altered by CTSB- knockdown using different siRNAs (i.e. shCTSB1, shCTSB2).**

Uploaded as supplementary excel file.

**Fig S1: Pathways and Functional enrichment analysis of Resistance signature from 786-O and A498 RCC xenograft mouse model.**

A). Functional enrichment analysis. Each bar represents significantly affected functions in the indicated tumor cell xenograft. Magnitude of activation and inhibition of functional category is calculated on basis of Z-score and shown along X-axis. B) Pathways enrichment analysis. Each bar represents a significantly enriched pathway as determined using Fisher’s Exact Test p-value. P values are depicted as –log10 (p-value) on the X-axis. A). Functional enrichment analysis. Each bar represents significantly affected functions in the indicated tumor cell xenograft. Magnitude of activation and inhibition of functional category is calculated on basis of Z-score and shown along x-axis.

**Fig S2. CTSB expression in the 786-O RCC xenografts of LKO vs shCTSB2.**

A). Tumor lysates from mice in the 786-O model of LKO vs shCTSB2 confirm CTSB knockdown reduced CTSB expression by real-time PCR. B). Tumor lysates from mice in the 786-O model of LKO vs shCTSB2 confirm CTSB knockdown reduced CTSB expression by immunoblot.

**Fig S3.** **Enrichment map of Biological processes significantly (P value<.05) affected by CTSB knockdown.**

A) Biological processes affected by downregulated genes. (B) Biological processes affected by upregulated genes. Each node of the enrichment map represents a biological process, and each line represents a group of common enriched genes between the connected biological processes.

**Fig S4. Gene expression and pathway enrichment analysis shows that CTSB knockdown reduces abundance of gene products related to the stem cell phenotype.**

Real-time PCR from mouse tumor samples reveals that CTSB knockdown reduced ALDH1 mRNA expression nearly completely in the 786-O model LKO vs shCTSB2. B). Pathways analysis on differentially expressed genes identified multiple metabolic pathways (P value <0.05) including "Chondroitin Sulfate Biosynthesis", "Eicosanoid Signaling", "Melatonin Degradation I”, "L-cysteine Degradation II", "Tight Junction Signaling", "CDK5 Signaling", and "Atherosclerosis Signaling". C). CTSB knockdown in the 786-O LKO, shCTSB1 and shCTSB2 cell lines leads to reduction of proteins related to the stem cell phenotype including, LIN28A, c-Myc, KFL4, Oct-4A, Sox2, ALDH1 and CTSB itself. D). CTSB knockdown in 786-O cells reduced mRNA expression of ALDH1, CD44, CXCR4, Nanog, Oct4, ALDH1 and VEGF, as assessed by real-time PCR.

**Fig S5. CTSB expression is not affected by sunitinib treatment *in vitro.***

786-O and A498 were treated with sunitinib (5 μM for 2 hours). The cells were lysed and examined by immunoblotting with antibodies as indicated.
